# Postkeratoplasty Anterior and Posterior Corneal Surface Wavefront Analysis: Descemet's Stripping Automated Endothelial Keratoplasty versus Penetrating Keratoplasty

**DOI:** 10.1155/2013/210565

**Published:** 2013-09-12

**Authors:** Maria L. Salvetat, Marco Zeppieri, Flavia Miani, Paolo Brusini

**Affiliations:** Department of Ophthalmology, Santa Maria della Misericordia University Hospital, Piazzale S. Maria della, Misericordia 15, 33100 Udine, Italy

## Abstract

*Purpose*. To compare the higher-order aberrations (HOAs) due to the anterior and posterior corneal surfaces in patients that underwent either Descemet-stripping-automated-endothelial-keratoplasty (DSAEK) or penetrating keratoplasty (PK) for endothelial dysfunction and age-matched controls. *Methods*. This retrospective, observational, case series included 28 patients after PK, 30 patients after DSAEK, and 30 healthy controls. A Scheimpflug imaging system was used to assess the HOAs due to the anterior and posterior corneal surfaces at 4 mm and 6 mm optical zones. Total, 3rd and 4th order HOAs were considered. Intra- and intergroup differences were assessed using the Friedman and the Kruskal-Wallis tests, respectively; paired comparisons were performed using Duncan's multiple range test. *Results*. Total, 3rd and 4th order HOAs due to both corneal surfaces at 4 mm and 6 mm optical zones were significantly higher in the PK group, intermediate in the DSAEK group, and lower in controls (*P* < 0.05). The most important HOAs components in both PK and DSAEK groups were trefoil and coma from the anterior corneal surface (*P* < 0.05) and trefoil from the posterior corneal surface (*P* < 0.05). *Conclusions*. The optical quality of both corneal surfaces appeared significantly higher after DSAEK than after PK, which can increase the postoperative patient's quality of vision and satisfaction.

## 1. Introduction

Endothelial keratoplasty (EK) is nowadays considered as the procedure of choice for the treatment of the endothelial dysfunctions [1]. The technique is based on the selective replacement of diseased endothelium, while leaving the healthy recipient anterior cornea structurally intact. EK has been shown to be a better procedure than penetrating keratoplasty (PK) due to faster postoperative visual recovery, minimal induced topographic changes, lower refractive error, higher refraction predictability and stability, absence of suture-related complications, better corneal structural integrity and innervation maintenance, and reduced risk of graft rejection [2–4]. The surgical technique has undergone modifications and improvements over the years, which include the following methods in chronologic order: *posterior lamellar keratoplasty* (PLK) [5],* deep lamellar endothelial keratoplasty* (DLEK) [6], *Descemet stripping endothelial keratoplasty* (DSEK) [7], *Descemet stripping and automated endothelial keratoplasty* (DSAEK) [8–10], and *Descemet membrane endothelial keratoplasty* (DMEK) [11].

The DSAEK technique, which currently tends to be the preferred EK surgical approach used in many centers, involves the mechanical stripping of the diseased host endothelium and Descemet's membrane and replacement with a donor graft, composed of endothelium, Descemet's membrane, and a thin layer of posterior stroma, carried out with an automated microkeratome [8–10].

Although several studies have shown higher postoperative visual outcomes after DSAEK than after PK [3, 12, 13], others have reported that the best spectacle-corrected visual acuity (BSCVA) after DSAEK can be lower than after PK [10, 14]. Patient's age, preoperative corneal haze, interface haze, and optical irregularities at the corneal surfaces and/or interface, inducing light scattering and increased irregular astigmatism, are suggested reasons for limited visual outcomes after DSAEK [14–20].

Several studies have shown that the DSAEK procedure causes minimal changes of the anterior corneal surface, with consequent optical advantages compared to PK such as lower regular [3, 10, 12] and irregular astigmatism, also known as higher-order aberrations (HOAs) [16–20]. Induced irregular astigmatism of the posterior corneal surface has shown to be either comparable between PK and DSAEK [17, 19, 20] or higher after DSAEK than after PK [16–18].

The wave-front analysis is an objective method of assessing the optical quality of the ocular refractive surfaces, by the evaluation of the low- and higher-order aberrations that can degrade the retinal image [21]. Great amounts of HOAs, which are not correctable by conventional spectacles or soft contact lenses, has been shown to reduce the optical performance of the eye by inducing halos, glare, monocular diplopia, decreased contrast sensitivity, and visual acuity [22, 23], especially under mesopic or scotopic conditions [24]. The cornea is the main contributor of HOAs in the eye and, regardless of cause, corneas with increased wavefront error show significant decreases in visual performances that are pupil size dependent [25].

The rotating Scheimpflug imaging system is a relatively new noncontact method that is able to provide highly repeatable measurements of the anterior and posterior corneal curvatures, which can be converted into corneal aberrations measurements by the device software [26, 27].

The aim of our study was to compare the HOAs due to the anterior and posterior corneal surfaces evaluated with a Scheimpflug-based corneal topographer in patients that underwent DSAEK or PK for endothelial dysfunction and age-matched controls with normal corneas, in order to assess the effect of PK and DSAEK on the optical quality of the corneal surfaces. Measurements were taken for a 4 mm and 6 mm optical zone, to simulate the photopic and scotopic conditions, respectively.

## 2. Patients and Methods

This retrospective, observational, and comparative case series study included 3 groups of subjects: 30 consecutive patients after PK, 31 consecutive patients after DSAEK, and 30 age-matched healthy subjects with normal corneas (control group). One eye per patient was considered. The study was in compliance with the tenets of the Helsinki's Declaration, and informed consent was obtained from all participants prior to testing. Each participant underwent the following examinations on the same day: complete ophthalmologic examination, including a review of medical history, BSCVA measured using the Snellen VA chart, manifest refraction evaluation (including spherical equivalent and cylindrical error), slit-lamp examination, fundus biomicroscopy with a 90D lens, Goldmann applanation tonometry measurement, and imaging with a Scheimpflug-based corneal topographer (Sirius 3D, CSO, Florence, Italy).

Normal subjects were recruited from staff members and volunteers. PK and DSAEK patients were recruited from the Cornea Clinic of the Department of Ophthalmology at S. Maria della Misericordia Hospital, Udine, Italy. The study was in compliance with Institutional Review Board (IRB) and HIPAA requirements and approved by the IRB of S. Maria della Misericordia Hospital, Udine, Italy.

Normal eyes were defined as no ocular disorders except for refractive errors, normal cornea appearance, normal corneal topography results, no history of ocular surgery, no previous corneal or conjunctival disease that is likely to affect the corneal HOAs, and no family history of ocular pathologies.

Inclusion criteria for postoperative patients were previous PK or DSAEK for endothelial dysfunction (at least 6 months after complete suture removal); availability of postoperative Scheimpflug camera imaging with no missing data points within the central 6.0 mm zone, no intra- or postoperative complications that can affect the Scheimpflug camera measurements, no history of ocular surgery other than cataract surgery, and willingness to provide informed written consent. Exclusion criteria included corneal scars or opacities of the graft, history of postoperative ocular infection or trauma, graft rejection, history of intraocular surgery other than keratoplasty or cataract surgery, presence of ocular or systemic diseases or medications that could affect the ocular surface and/or prevent reliable wave-front measurements, and inability to comply with Scheimpflug imaging procedure.

PK [3] and DSAEK [8, 10] techniques have extensively been reported elsewhere and have briefly been described in the Appendix. All surgeries were performed by a single surgeon (PB) from June 2008 to March 2010 at the Department of Ophthalmology of the S. Maria della Misericordia Hospital, Udine, Italy. Corneal diseases requiring keratoplasty were Fuchs dystrophy and pseudophakic bullous keratopathy. The mean duration of the bullous keratopathy was 9.1 ± 9.3 months (range 6 to 43 months). Donor corneas in the form of a sclerocorneal button stored in organ culture at 31°C were provided by the “Fondazione Banca degli Occhi del Veneto” (Venezia-Mestre, Italy) Eye Bank. In 6 patients with significant lens opacity, 2 PK and 4 DSAEK patients, standard phacoemulsification was performed using the phaco chop technique, followed by implantation of an intraocular lens (IOL) in the bag.

BSCVA in Snellen lines and refraction, reported as mean refractive spherical equivalents, were measured by a single optometrist (LP), who was masked to the type of surgery.

The Sirius 3D rotating Scheimpflug camera (CSO, Florence, Italy) was used for all corneal measurements. This noncontact instrument combines a rotating Scheimpflug camera with a Placido disk technique, providing high-resolution images of the anterior segment, anterior and posterior corneal topography, and pachymetry of the entire cornea. The system uses a rotating Scheimpflug camera and a monochromatic slit-light source that rotate together around the optical axis of the eye for 180 degrees and acquires 25 to 50 images from the anterior segment, allowing the acquisition of anterior and posterior corneal elevation topographic data. The built-in software provides the conversion of the corneal elevation profile into corneal wave-front data using the Zernike vector terms [28] with an expansion of up to the 10th order. The root mean square (RMS) of the Zernike vector magnitude is calculated and expressed in *μ*m. Data from an area of up to 10 mm in diameter are provided by the instrument; a graft size of 8.5 mm was considered in our study. The automatic release mode was used, which achieves correct focus and alignment with the corneal apex before scanning starts. The imaging was performed with the patient seated and correctly positioned in the chinrest and forehead strap. The patient was asked to keep both eyes open and to look at a fixation target. The system constantly monitors eye movements; measurements with decentration less than 0.6 mm are considered valid. The examination quality data were assessed with a built-in program, and the results with significant errors were excluded. Three measurements were taken by the same experienced examiner (LP) from each eye, and the best scan with the less distorted Scheimpflug image was used for analysis. All measurements were collected at the last visit and at least 6 months after complete suture removal for postkeratoplasty eyes. The following measurements were considered: simulated keratometric (simK) values in the 3 mm central zone, RMS of the Zernike vector magnitude of the total HOAs, and 3rd and 4th order aberrations of the anterior and posterior corneal surface within the central 4 mm and 6 mm zones. Total HOAs were defined as the sum of the magnitude of the Zernike vector terms of 3rd to 7th order. SimK values include diopter power and axis of the steepest meridian and at 90 degrees (K1 and K2). K1 and K2 were averaged to obtain a single corneal curvature value. The corneal astigmatism value was defined as the absolute value for K2 minus K1. 

Data were analyzed using the statistical analysis software SPSS for Windows, version 20.0 (SPSS Inc., Chicago, IL, USA). Data were described by medians (standard deviation) and 95% confidence interval (CI). Normality of the data distribution was assessed with the Kolmogorov-Smirnov test. Intragroup differences were assessed using the Wilcoxon and Friedman tests; intergroup differences were calculated using the Kruskal-Wallis tests; the Duncan multiple range test was used for multiple comparisons. Correlations were tested using the Spearman's rank correlation coefficient. Statistical significance was defined as *P* < 0.05.

## 3. Results

**Figure 1:**
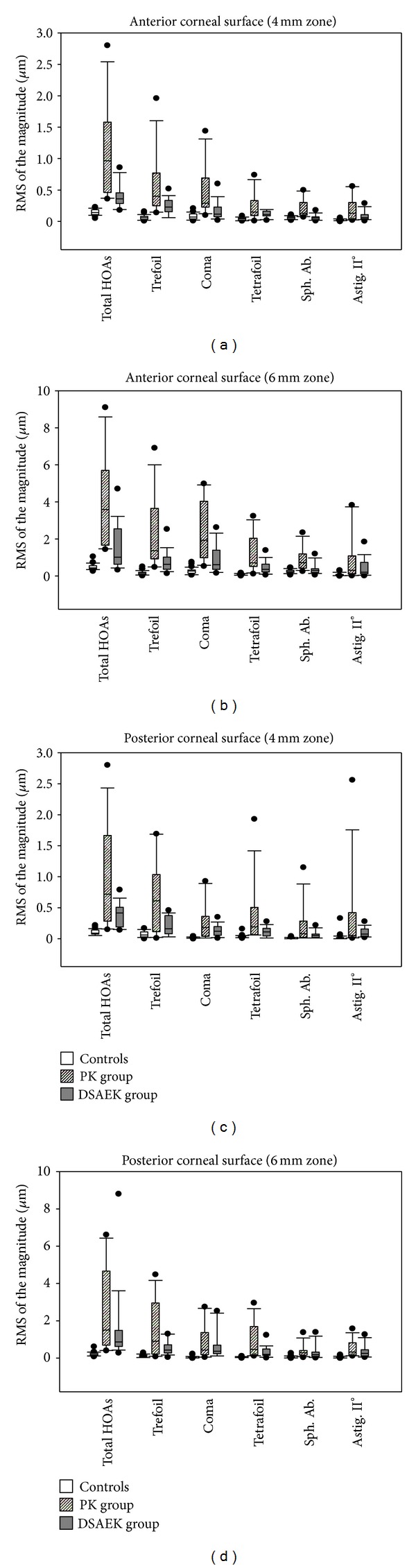
Box-plot representation of the magnitude (expressed as RMS in *μ*m) of the total, 3rd and 4th order HOAs due to the anterior corneal surface within the 4 mm (a) and 6 mm (b) central zones, and due to the posterior corneal surface within the 4 mm (c) and 6 mm (d) central zones, in the control, PK and DSAEK groups. The magnitude of the spherical aberration was expressed as absolute value. Median values are represented as dark lines, 25/75 percentiles as boxes, 5/95 percentiles as bars, and outliers as circles.

Scheimpflug imaging was not obtained for 2 patients after PK and 1 patient after DSAEK and thus were excluded from the analysis. A total of 28 patients after PK, 30 patients after DSAEK, and 30 healthy controls fulfilled the inclusion criteria. Detailed demographic, visual, and refractive characteristics of the three groups of subjects are listed in Table 1. The wavefront analysis of the corneal HOAs of the 3 groups is shown in Figure 1.

### 3.1. Anterior Corneal Surface (Figures 1(a) and 1(b))

Total HOAs, trefoil, and coma were significantly higher in the PK group, intermediate in the DSAEK group, and lower in controls (*P* < 0.05). Tetrafoil appeared significantly lower in controls (*P* < 0.01), comparable between PK and DSAEK eyes within the 4 mm zone (*P* > 0.05), and significantly higher in the PK eyes than in DSAEK eyes within the 6 mm zone (*P* < 0.05). Spherical aberration and secondary astigmatism were significantly higher in the PK group than in the other groups (*P* < 0.05).

The most important aberration components were trefoil, coma, and spherical aberration in controls (*P* < 0.05), and trefoil and coma in the DSAEK and PK groups (*P* < 0.05).

### 3.2. Posterior Corneal Surface (Figures 1(c) and 1(d))

Total HOAs, trefoil, coma, and tetrafoil were significantly higher in the PK eyes, intermediate in the DSAEK eyes, and lower in controls (*P* < 0.05). Spherical aberration and secondary astigmatism appeared significantly lower in controls (*P* < 0.01), higher in the PK than in the DSAEK eyes within the 4 mm zone (*P* < 0.05) and comparable between PK and DSAEK groups within the 6 mm zone (*P* > 0.05).

The trefoil was the most important aberration component within the 4 mm zone in the three groups (*P* < 0.01). The most important aberration components within the 6 mm zone were trefoil in the control and PK eyes (*P* < 0.05), and coma and trefoil in the DSAEK eyes (*P* < 0.05).

The magnitude of the HOAs from the 4 mm zone was significantly lower than that of the HOAs from the 6 mm zone for both corneal surfaces in all groups (*P* < 0.01). In controls, the magnitude of the HOAs of the anterior corneal surface was significantly larger than that of the posterior surface (*P* < 0.01), with exception of that of trefoil, tetrafoil, and secondary astigmatism within the 4 mm zone, which was comparable between corneal surfaces (*P* > 0.05).

**Figure 2:**
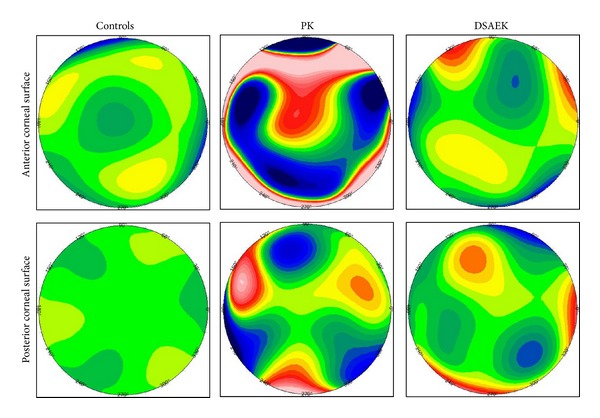
Representative wavefront maps of anterior and posterior corneal surfaces in normal, PK, and DSAEK eyes. The same color scale was used for all maps to allow comparisons.

In the PK and DSAEK groups, the magnitude of the HOAs of the corneal anterior surface appeared comparable with that of the posterior corneal surface (*P* > 0.05), except for that of the total HOAs and coma within the 6 mm zone, which appeared to be significantly higher on the anterior surface (*P* < 0.05). Representative aberration color-coded maps of anterior and posterior corneal surfaces within the 6 mm central zone in normal, PK and DSAEK eyes are shown in Figure 2. The correlations between the BSCVA and the magnitude of the HOAs of both corneal surfaces were not statistically significant in any of the 3 groups (*P* > 0.05).

## 4. Discussion

The results of our study showed that the magnitude of the total HOAs and of the Zernike vector terms of 3rd and 4th order from both corneal surfaces was significantly higher in PK and DSAEK eyes than in controls, indicating greater corneal surface irregularities in grafted eyes. In accordance with our results, several previous authors have reported a greater amount of HOAs from both corneal surfaces in PK eyes in comparison with normal eyes [16, 17, 19, 20, 29, 30]. The asymmetric distortion of both corneal surfaces induced by differences in curvature, thickness, and diameter between donor lenticule and recipient bed, in addition to the wound configuration induced by the healing process, may explain the increased amount of corneal HOAs found after PK.

The magnitude of the anterior corneal HOAs was significantly higher after DSAEK than in controls in our study, which is in disagreement with previous authors that did not find significant differences between normal and DSAEK eyes [16, 17, 20] and in accordance with others [19]. This may be explained by the corneal incisions and related wound healing after the DSAEK procedure. Several other studies [16, 17, 19, 20], however, have reported that the posterior corneal HOAs appeared significantly lower in controls than in DSAEK eyes, due to the insertion of the donor lenticule that induces evident configuration changes of the corneal posterior surface [31, 32].

The comparison between PK and DSAEK eyes showed that the magnitude of the total, 3rd and 4th order HOAs from both corneal surfaces, within both optical zones of 4 mm and 6 mm, was significantly higher in PK than in DSAEK eyes. These data suggest a higher optical quality of the corneal surfaces after DSAEK than after PK.

In agreement with our data, several previous authors have reported significantly greater ocular and anterior corneal surface HOAs after PK than after DLEK [29, 33], DSAEK [16–20], and DMEK [20] procedures. The ocular HOAs have been demonstrated to be significantly higher after DLEK than after DSAEK, which could be related to the rougher surface created by the hand dissection of the donor and recipient corneas, inducing higher interface irregularities [34]. Moreover, the corneal anterior HOAs have been reported to be minimal and comparable after DSAEK and DMEK procedures [20], suggesting that both surgical procedures induce only slight changes in the anterior corneal configuration.

The comparison regarding the amount of the posterior corneal surface HOAs between PK and DSAEK surgery is still a debatable issue. Our data showed greater posterior corneal surface HOAs after PK than after DSAEK. In disagreement with our results, studies have reported that the HOAs due to the posterior corneal surface were either comparable between the two groups [17, 19, 20] or higher after DSAEK than after PK [16, 18]. The different results found in our study in comparison with those reported by previous authors can be related to differences in the cohort of patients, type of surgical procedures, diameter of optical zone considered, and devices used to measure the corneal wave-front errors. Previous studies have reported that values provided by the Pentacam system for posterior corneal aberrations in normal subjects were likely to be erroneous [35]. The new Scheimpflug-based topographer used in our study may provide a more accurate evaluation of the corneal HOAs.

As found in our study, the contribution of the anterior surface to the corneal HOAs tended to be significantly higher than that of the posterior surface, due to differences in refraction indices [36]. The impact of the posterior corneal surface on vision, however, has yet to be sufficiently explained. Although previous authors found that VA correlated significantly with the HOAs due to the anterior corneal surface but not with those from the posterior corneal surface in normal and postkeratoplasty eyes [30, 37], others suggested the possible influence of posterior corneal curvature on visual function [38]. A recent study comparing VA and corneal HOAs between DSAEK and DMEK eyes showed that a significantly higher postoperative BSCVA in the DMEK eyes was associated with comparable corneal HOAs from the anterior surface and significantly higher HOAs from the posterior surface in the DSAEK eyes [20], suggesting a relationship between visual function and posterior corneal surface regularity.

In both PK and DSAEK groups, the most important aberration components were trefoil and coma from the anterior corneal surface and trefoil from the posterior corneal surface. These data suggest that both surgical procedures induce an increased surface irregularity of the entire cornea at the anterior surface and a greater peripheral corneal configuration distortion at the posterior corneal surface.

The prevalence of trefoil after PK has already been reported [12, 16] and may be explained by wound malapposition brought on by irregular wound incisions and differences in donor-host graft diameters, which can cause peripheral local deformations of the graft. Coma and spherical aberration, also found in PK eyes [16, 33], have been considered as the result of a slight decentration of the donor cornea and of a midperipheral cornea relaxation, induced by the wound, with consequent steepening of the central cornea, respectively.

The prevalence of coma and trefoil from both corneal surfaces in DSAEK eyes has been found by other authors [16]. These results could be related to the corneal incisions and related wound healing for the anterior surface and to a slight decentration and/or irregularity of the peripheral portion of the donor lenticule for the posterior one. Experimental studies have demonstrated that each Zernike term has a different impact on vision and that spherical aberration and coma are the most visually significant aberrations and can have a detrimental effect on vision also at small pupil size [39, 40]. Considering that the HOAs of both corneal surfaces (especially coma and spherical aberration) were significantly lower in the DSAEK than in PK eyes within both optical zone of 4 mm and 6 mm, the visual performance could be much more impaired after PK than after DSAEK procedure, either under photopic or scotopic conditions. In accordance with previous authors [33] and in disagreement with others [18, 30], we did not find any correlation between BSCVA and the magnitude of the total, 3th and 4th order HOAs of both corneal surfaces in normal and postkeratoplasty eyes. The lack of significant relationship between the BSCVA and HOAs magnitude can be due to the presence of ocular pathologies other than endothelial dysfunction in DSAEK and PK eyes (especially cataract and macular degeneration) and the low sensitivity of the photopic high-contrast VA to limited wave-front errors variation, especially when acuity is scored to line as opposed to the letter in normal eyes, as was the case in our paper [21, 40].

Our study has several limitations, including that it was based on retrospective data, the number of eyes considered was relatively small, and the instrument reproducibility was not assessed. Previous studies, however, have reported that anterior and posterior corneal curvature parameters assessed by the rotating Scheimpflug camera were highly repeatable [26]. Another limitation is the fact that only the high-luminance high-contrast BSCVA was evaluated, which has shown to be less sensitive to HOAs variation than contrast sensitivity and low-contrast visual acuity under mesopic and scotopic conditions [21, 40]. Moreover, the correlation between BSCVA and HOAs magnitude was unsurprisingly not significant considering that both PK and DSAEK patients could be affected by ocular pathologies other than endothelial dysfunction. Our previous study [41] showed that the total HOAs from the anterior corneal surface were significantly lower in DALK than in ALTK and PK groups; however, the total HOAs from the posterior corneal surface were comparable amongst postoperative groups. The aberration components that were significantly greater included coma in the KC and ALTK eyes, trefoil and coma in the DALK eyes, and trefoil in the PK eyes. Further studies are currently underway, which include only patients after PK and DSAEK without any other ocular disease (with exception to pseudophakia).

In conclusion, the measurement of the corneal wave-front errors can be important in understanding the changes induced by penetrating or lamellar keratoplasty on the corneal profile. The results of our study showed that the DSAEK procedure can provide a higher postoperative regularity to both corneal surfaces when compared with PK. This can lead to better postoperative corneal optical quality, thus providing enhanced patient visual performance and satisfaction. Further studies evaluating contrast sensitivity and low-contrast visual acuity under mesopic and scotopic conditions are needed to better assess the influence of the corneal wave-front error on the visual quality after PK and DSAEK surgery.

## Figures and Tables

**Table 1:** Demographic and refractive data.

	Controls	PK group	DSAEK group	Comparison
	(*n* = 30)	(*n* = 28)	(*n* = 30)	(P*)
Patient's age (years)	66.6 ± 15.7	67.7 ± 14.1	70.5 ± 12.4	0.10
	(40–87)	(21.8–86.2)	(43.2–84.8)	
Time interval from surgery (months)	—	33.3 ± 14.6	32.5 ± 13.1	0.58
		(19.1–47.3)	(18.7–43.1)	
Time interval from suture removal (months)	—	23.9 ± 13.8	26.0 ± 12.4	0.43
		(11.2–35.1)	(10.4–34.9)	
BSCVA (Snellen lines)	0.91 ± 0.2^a^	0.53 ± 0.3	0.60 ± 0.2	0.001
	(0.5–1.0)	(0.1–1.0)	(0.4–1.0)	
Spherical-equivalent error (D)	0.05 ± 2.0	−1.45 ± 4.8^b^	0.33 ± 1.7	0.01
	(−3.7/2.5)	(−14.1/1.6)	(−2.1/3.9)	
Mean anterior corneal curvature (D)	43.9 ± 1.4	45.4 ± 2.8^a^	43.1 ± 1.6	0.008
(Sim K1 + Sim K2)/2	(41.1–47.5)	(42.1–51.4)	(39.3–45.8)	
Anterior corneal astigmatism (D)	0.75 ± 0.5	4.49 ± 2.9^a^	1.38 ± 0.5	0.0001
(Sim K2 − Sim K1) absolute value	(0.2–2.5)	(1.2–9.8)	(0.6–2.7)	

Results are given as median ± SD (95% confidence interval).

*Kruskal-Wallis test.

BSCVA: best spectacles-corrected visual acuity; D: diopters; Sim K: simulated keratometric value.

^a^Significantly higher than the other groups.

^b^Significantly lower than the other groups.

## APPENDICES

### A. Surgical Technique

Local anesthesia and akinesia were achieved with a peribulbar injection of 10 cm^3^ of a 1 : 1 mixture of bupivacaine 0.75% and lidocaine 2%.

### B. Penetrating Keratoplasty (PK)

A Hanna suction trephine (Moria, Antony, France) was used to cut a partial depth, circular incision in the recipient cornea, centered at the geometric center of the cornea, with a diameter of 8.25 mm. Excision of the recipient corneal button was completed with curved corneal scissors. A 0.25 mm oversized donor button was punched from the endothelial side with the Hanna punch trephine. Four interrupted sutures and a single 16-bite 10-0 nylon suture (Ethicon Inc., Somerville, NJ) were placed in all cases.

### C. Descemet Stripping Automated Endothelial Keratoplasty (DSAEK)

The donor lamellar graft dissection was performed with a hand-driven microkeratome using the Moria ALTK microkeratome (model Evolution 3E) equipped with a 350-micron-deep blade and associated artificial anterior chamber (Moria, Antony, France). After dissection, the anterior corneal cup was discarded, and the posterior corneal lamellar tissue was placed in the corneal storage medium Optisol (Chiron Ophthalmics, Irvine, CA). At the beginning of surgery, the posterior corneal lamellar tissue was transferred to a punching system and was punched from the endothelial side using an 8.5 mm Hanna punch trephine (Moria, Antony, France). The donor corneal lenticule remained resting on the donor punching block covered by Optisol solution until use. A clear corneal temporal incision was made in the host with a 2.75 mm keratome. The recipient epithelium was marked with an 8.5 mm Weck trephine (Solan Medtronics, Jacksonville, FL) stained with gentian violet dye to outline where to strip the Descemet membrane and to place the donor tissue. Two paracenteses were made at the 7- and 10-o'clock positions. A paracentesis was made 2 hours clockwise from the corneal incision to allow the positioning of an AC maintenance cannula. The host endothelium and the Descemet membrane were scored in a circular pattern under the area of the epithelium mark for a diameter of 8.5 mm, using a reverse-bent Price-Sinskey hook (Asico, Westmont, IL). The Descemet membrane and the endothelium were stripped using a Price hook and spread on the anterior surface of the recipient cornea to make sure a sufficient area had been removed. The clear corneal incision was widened to approximately 4.2 mm using the keratome. The donor corneal lenticule was placed on a Busin-glide (Moria USA, Doylestown, Pennsylvania, USA) (endothelial side up) and inserted into the AC using the Price forceps. Unfolding and positioning of the donor lamella were performed using air carefully inserted in the CA with a 30-gauge cannula, and a Sinskey hook was used to match the donor within the recipient dissection edges. After the AC was filled with air for 7–10 minutes, part of the air was removed and replaced with balanced salt solution (BSS).

After surgery, all patients underwent patching overnight. DSAEK patients were instructed to lie supine for at least 6 hours. Beginning the next morning, 0.1% dexamethasone sodium phosphate and ofloxacin (Alcon Laboratories, Inc. Fort Worth, TX, USA) eye drops were administered 4 times daily for 1 week. The antibiotic drops were discontinued 1 week after surgery, and dexamethasone eye drops were tapered for 12 months in all groups. In PK eyes, the running suture was removed 12 to 18 months after surgery.
